# Cellular infiltration in traumatic brain injury

**DOI:** 10.1186/s12974-020-02005-x

**Published:** 2020-11-03

**Authors:** Aftab Alam, Eric P. Thelin, Tamara Tajsic, Danyal Z. Khan, Abdelhakim Khellaf, Rickie Patani, Adel Helmy

**Affiliations:** 1 Division of Neurosurgery, Department of Clinical Neurosciences, University of Cambridge, Cambridge, UK; 2 Department of Clinical Neuroscience, Karolinska Institutet, Stockholm, Sweden; 3 Department of Neurology, Karolinska University Hospital, Stockholm, Sweden; 4 Injury Prevention Research Office, Division of Neurosurgery, St. Michael’s Hospital, University of Toronto, Toronto, Canada; 5 Department of Neuromuscular Diseases, Queen Square Institute of Neurology, University College London, London, UK; 6 The Francis Crick Institute, London, UK

**Keywords:** Neuroinflammation, Cellular infiltration, Traumatic brain injury, Microglial dynamics

## Abstract

Traumatic brain injury leads to cellular damage which in turn results in the rapid release of damage-associated molecular patterns (DAMPs) that prompt resident cells to release cytokines and chemokines. These in turn rapidly recruit neutrophils, which assist in limiting the spread of injury and removing cellular debris. Microglia continuously survey the CNS (central nervous system) compartment and identify structural abnormalities in neurons contributing to the response. After some days, when neutrophil numbers start to decline, activated microglia and astrocytes assemble at the injury site—segregating injured tissue from healthy tissue and facilitating restorative processes. Monocytes infiltrate the injury site to produce chemokines that recruit astrocytes which successively extend their processes towards monocytes during the recovery phase. In this fashion, monocytes infiltration serves to help repair the injured brain. Neurons and astrocytes also moderate brain inflammation via downregulation of cytotoxic inflammation. Depending on the severity of the brain injury, T and B cells can also be recruited to the brain pathology sites at later time points.

## Background

Traumatic brain injury (TBI) results from force transmission to the head either by impact with an object or from acceleration/deceleration forces that produce vigorous movement of the brain within the skull or varying combinations of these mechanical forces [1]. It is now widely acknowledged that TBI is a multimodal complex disease process and not a single pathophysiological event [2]. It triggers structural and functional changes leading to neuronal injury which can be classified into primary and secondary brain injury [3]. Primary injury is caused by external forces (direct contact and/or inertial forces to the brain) acting at the moment of the injury that damages the blood vessels, axons, nerve cells, and glia of the brain in a focal, multifocal, or diffuse pattern of involvement. The type and severity of the resulting injury depends upon the nature of the original force plus the site, direction, and magnitude of the force [4]. In contrast, secondary injury is an ongoing process that occurs from minutes to years following the initial insult. Secondary injury is the result of the cascades of metabolic, neurochemical, cellular and molecular events stemming from primary insult [5]. Such mechanisms ultimately lead to brain cell death, plasticity, tissue damage and atrophy [6–8]. Some of the biochemical alterations responsible for secondary injury are (i) perturbation of cellular calcium homeostasis, (ii) glutamate excitotoxicity, (iii) mitochondrial dysfunction, (iv) increased free radical generation, (v) inflammation, (vi) increased lipid peroxidation, (vii) apoptosis, (viii) diffuse axonal injury (DAI) and (ix) blood-brain barrier breakdown [9]. Of note, all of the above-listed factors can be linked to neuroinflammation either directly or indirectly, and such inflammation has been implicated in both the early and chronic components of TBI-induced neuropathology [10–12].

Damaged neuronal tissue releases chemokines which in turn recruit immune cells to the area of injury [13, 14]. Depending on the nature of the insult, traumatic contusion, diffuse injury or raised intracranial pressure can contribute to the resulting inflammatory response, with potentially distinct cellular patterns. In focal injury (experimental animal models), the infiltration of neutrophils appears early peaking within a few days followed by the migration of microglia/macrophages, astrocytes and lymphocytes to the site of injury [15]. In a focal drop device model in rats, Al Nimer et al. reported a 10- to 20-fold increase in the numbers of microglia as compared to the peripheral macrophages, suggesting the occurrence as a central rather than peripheral response [16]. In diffuse injury, preclinical studies report little to no neutrophil infiltration, with the early cellular response consisting of microglial accumulation and astrocytosis most prominent in the white matter tracts [17]. In a recent study, we observed differential downstream production of cytokines in enriched human cortical neuronal cultures exposed to similar levels of cytokines seen in the brain following human TBI, which may modify pathophysiological processes in both beneficial and detrimental ways in the in vivo situation. Our finding suggests that IL (Interleukin)-1β induced a limited cytokine response in neurons which may echo a relative paucity of IL-1 receptors (IL-1r) on the neuronal population [18]. In a follow-up study using a similar approach, enriched iPSC (induced pluripotent stem cells)-derived astrocytic cultures exposed to escalating concentrations of different cytokines generate an increased production of downstream cytokines, particularly when exposed to IL-1β [19].

Following TBI, the cytokine response comes from the diverse immunocompetent cell types such as microglia, astrocytes, cerebrovascular endothelial cells, peripheral immune cells, and even neurons. As such, the inflammatory response is signalled by a rapid rise in cytokines and chemokines [20–23]. After a moderate diffuse-TBI in mice, levels of typical pro-inflammatory cytokines such as IL-1β, tumour necrosis factor (TNF) and IL-6 within the cortex peak at 3-9 h before gradually diminishing [24]. Similarly, within clinical studies, increased levels of IL-6, TNF, IL-10, C-C motif chemokine ligand 2 (CCL2) and IL-8 peak within the first 2 days following brain injuries and then return to normal over several weeks [23, 25, 26]. This spike in cytokine release has been correlated with astrogliosis, microglial activation and axonal dysfunction, providing evidence of the association between the activated immune response and brain pathology [27]. We have reported both a dose- and/or time-dependent release of cytokines in our enriched human neuronal culture model of injury [19]. The IL-6 and TNF exposure each resulted in significantly increased levels of more than 10 cytokines over time, whilst IL-1β increased the level of C-X-C motif chemokine-10 (CXCL10/IP10) alone. Importantly, these patterns are consistent with our previous in vivo human TBI data [28], thus validating our human stem cell-derived neuronal platform as a clinically useful reductionist model. In the present review, we will discuss the role of cellular infiltration in inflammation after TBI.

## Mechanisms of cellular infiltration after TBI

### DAMPs and innate immunity

**Fig. 1:**
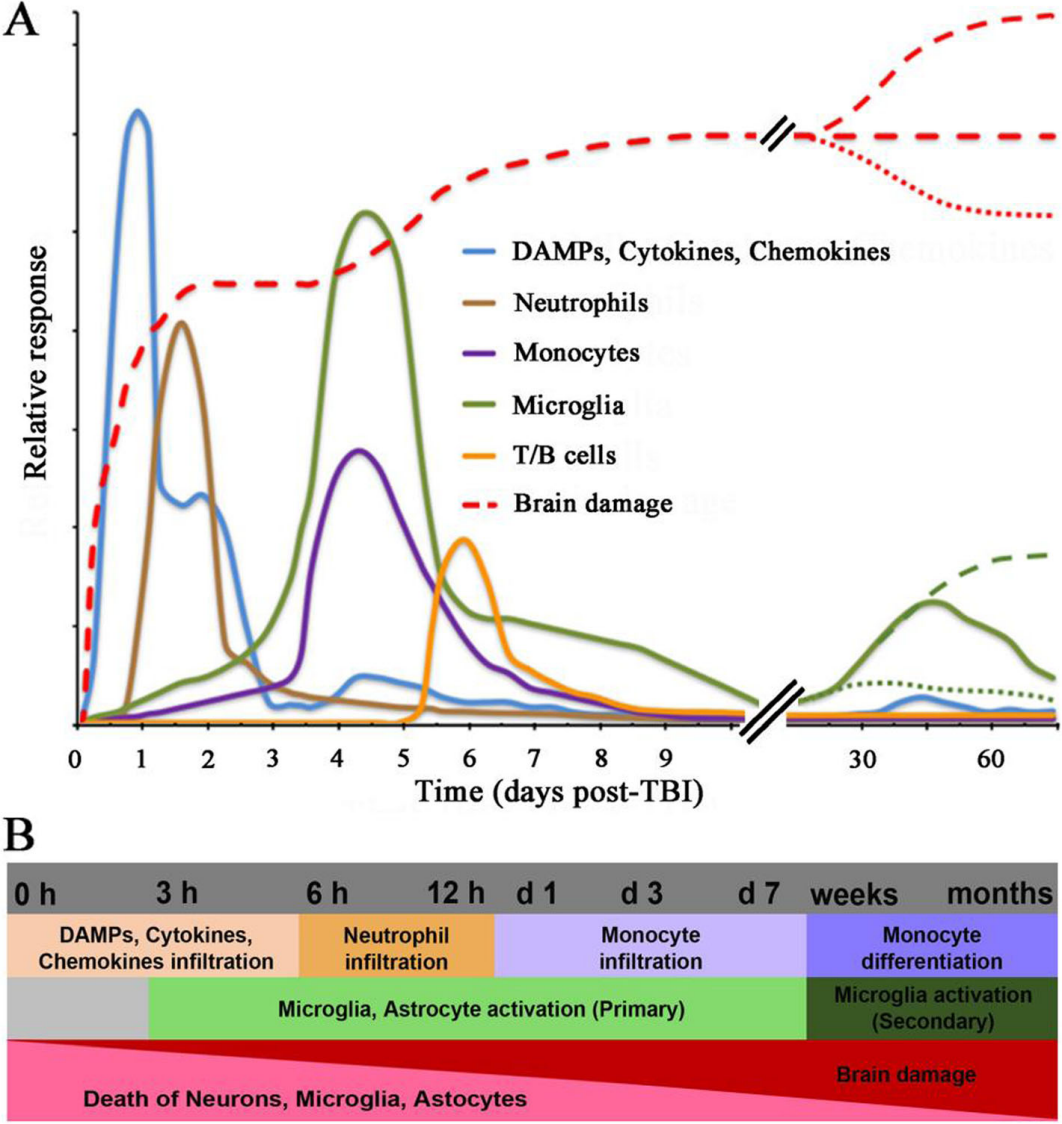
Timeline of cellular response after TBI. **a** Upon TBI, cellular damage results in the rapid release of damage-associated molecular patterns (DAMPs) that prompt resident cells to release cytokines and chemokines. These signals quickly recruit neutrophils, which aid in the containment of the injury site and promote the removal of debris and damaged cells. As neutrophil numbers begin to decline after days, infiltrating monocytes and activated microglia and astrocytes begin to accumulate around the site of injury to perform reparative functions. Depending on the severity of the brain injury, T and B cells can also be recruited to sites of brain pathology at later time points in the response (5-7 days post-injury). Severity of the brain damage (red dashed) depends on the secondary injury mainly caused by the fluctuating microglia especially the M1-type (neurotoxic) microglia as shown by green dashed lines. **b** Schematic representation to highlight the behaviour of cells after TBI

The innate immune system is a complex network of cells and signalling mediators that serves as the first line of defence against invading pathogens and injuries. In the case of TBI where the initial mechanical trauma causes direct damage, it is the microglia and astrocytes that initiate a cascade of immune events directed by the release of damage-associated molecular patterns (DAMPs) [29, 30]. The stimulation of nonspecific immune responses results in the recruitment of mononuclear phagocytes (MP; peripheral monocyte-derived macrophages and neutrophils) into the brain from the blood whilst activating resident glia (Fig. 1). These cells produce an array of pro-inflammatory mediators, reactive oxygen species (ROS) and pro-apoptotic proteins that perpetuate neural injury. The complexity of the innate immune response following TBI is demonstrated by the fact that more than one hundred genes related to inflammation and markers of microglia and astrocyte activation were significantly upregulated following controlled cortical impact (CCI) in mice [31].

### Key cytokines and chemokines

Cytokines are inflammatory regulators that are produced by systemic leukocytes, glial (astrocytes and microglia) cells and possibly by neurons [32]. These proteins (cytokines) are traditionally categorised as pro- or anti-inflammatory in action, although more recently this view has been nuanced, and they are now often classified into several families based on their receptor interactions. Many of these cytokines have been shown to play a role in brain injury and have been previously reviewed elsewhere [22]. When injury occurs to the brain, it activates the release and production of cytokines and chemokines which, in turn, trigger receptors resulting in local and systemic immune responses [33–35]. The net effect of these innate inflammatory mediators is aimed at limiting the spread of the injury and restoring homeostatic balance [36]. Here, we review five important cytokines/chemokines below that have been highlighted in the current TBI literature.

### Interleukin-1

The interleukin-1 family (IL-1 family) is a group of 11 cytokines playing a central role in the regulation of immune and inflammatory responses to infections or sterile insults. IL-1α, IL-1β, IL-1ra, IL-18, IL-33, IL-36α, IL-36β, IL-36γ, IL-36Ra IL-37 and IL-38 have been characterised in this group [37]. Interleukin-1 is primarily an active pro-inflammatory cytokine protein that has been associated with various inflammatory and neurological conditions and is likely one of the first immune mediator peaks just after the injury. Increased IL-1 production/secretion has been shown to aggravate neuroinflammation and neurodegeneration in the brain, therefore, production/secretion of IL-1 must be regulated firmly [38]. Within the IL-1 family, two distinct forms, IL-1α and IL-1β play a critical role in inflammatory signals following the engagement of IL-1 receptor (IL-1R). Both IL-1α and IL-1β, although isolated from two distinct complementary DNAs, induce almost identical downstream inflammatory responses but their activation requirements differ. IL-1α is essentially expressed by nucleated cells and discharged IL-1α can broadcast inflammatory signalling without further modification/processing. In contrast, IL-1β is produced in the form of a biologically inactive protein that needs cleavage to exhibit its inflammatory properties and secretion [39]. Levels of IL-1β have been shown to rise after brain injury and as such it is one of the most frequently measured cytokines in TBI research [40–53]. After brain injury, IL-1β is known to strongly enhance inflammatory responses and has profound effects on BBB (blood-brain barrier) permeability, glial activation, immune cell recruitment and ultimately in neurodegeneration [54–56]. To summarise, IL-1 production may negatively impact clinical outcomes following TBI [49].

Several recent methods have been trialled in human disease to neutralise/antagonise the activity of IL-1β in brain injury. Anakinra, a recombinant IL-1rreceptor antagonist (IL-1ra), is currently being tested to treat severe TBI in humans, as it has shown promise in the treatment of stroke by Emsley and coworkers [57]. Clausen and colleagues [58, 59] in their studies showed that the administration of anti-IL-1β neutralising antibody to CCI-injured animals for 14 days after TBI resulted in decreased numbers of macrophages/microglia, neutrophils and T-cells in the brain, especially at day 7 after injury. We have carried out a randomised controlled trial of Anakinra and utilised principal component analysis (PCA) to demonstrate that IL-1 signalling is a key upstream regulator of TBI-induced cytokines/chemokines production [60]. Later, we have also shown that recombinant IL-1ra promotes macrophages to express higher levels of pro-inflammatory cytokines such as granulocyte-macrophage colony-stimulating factor (GM-CSF) and IL-1β [61]. We demonstrated that treatment with Anakinra causes a brain-specific modifications of the cytokine/chemokine response in the first 48 h following injury [61]. The scale of this response is dependent on the IL-1ra concentration achieved in the brain extracellular fluid (ECF). In Anakinra-treated patients, chemokines related to recruitment of macrophages biassed towards M1-type microglia are increased. On the other hand, cytokines/chemokines biassed towards M2-type microglia are relatively increased in control patients. Against this background, it is clear that the simplistic classification of cytokines such as IL-1ra as an anti-inflammatory cytokine may not sufficiently capture the complexity of its role in neuroinflammation following brain injury.

### Interleukin-6

Interleukin-6 (IL-6), a 26 kDa glycoprotein, is a main effector of the acute phase response. Its receptor is a gp130 homodimer with a ligand-specific a-subunit. Binding of IL-6 induces dimerization of the receptor and activation of the JAK-STAT (Janus kinase-signal transducers and activators of transcription) intracellular pathway that regulates transcription of target genes within the cell nucleus through the nuclear translocation of phosphorylated STAT3 [62]. In humans, interleukin-6 has often been correlated with TBI outcome, but it is ambiguous whether its role is predominantly beneficial or detrimental. Brain ECF (extracellular fluid) detection of raised parenchymal IL-6 levels, using microdialysis, has been correlated with improved survival in brain injury patients [63]. Kumar and coworkers [50] showed that both subacute and chronic serum levels of IL-6 have correlated with unfavourable short and long-term consequences after TBI. In a follow-up study assaying IL-6 in human cerebrospinal fluid (CSF) and separating human patients by (high or low) CSF IL-6 trajectories, high trajectory patients were much more likely to have an adverse clinical aftermath [50]. Recent evidence by Liao et al. [64] in plasma suggests a detrimental role for IL-6 in human brain injury. These investigators showed that plasma levels of IL-6 were considerably higher in severe TBI patients than moderate TBI patients. The difference between these studies reflects the importance of recognising that distinct biological compartments (plasma vs CSF vs brain ECF) may represent distinct inflammatory responses and the additional contribution of extra-cranial injuries. Therefore, whilst a unitary role for IL-6 in brain injury across these compartments cannot be gleaned, evidence from TBI patients reveals that IL-6 is steadily upregulated after injury and can remain high in chronic stages, making it a potential mediator of long-term outcome.

### Tumour necrosis factor

Tumour necrosis factor (TNF) regulates many key processes in the central nervous system, including neuronal development, cell survival, synaptic plasticity, and ionic homeostasis. Furthermore, TNF acts as a major coordinator of the inflammatory response and dysregulated TNF signalling has been implicated in the pathophysiology of several CNS conditions, specifically relating to leukocyte infiltration [65] and increased BBB permeability [66]. In in vitro models, TNF acts synergistically with IL1 [67] and both are thought to act through similar intracellular transduction pathways [68].

Initial work on the role of TNF in TBI mouse models suggested that it has early harmful effects post-TBI whilst demonstrating more protective roles in chronic stages [69]. However, Stahel et al. [70] suggested that TNF is essential to protection from early mortality within the first week from injury. Irrespective of these seemingly contradictory studies, TNF consistently shows upregulation after brain injury, advocating an important role of TNF in both the acute and chronic stages [47, 49, 64, 71].

More recently, in a weight drop animal model, the importance of TNF early after injury was established. Baratz et al. [72] in their study demonstrated that mice that were given TNF inhibitor at 1 and 12 h post-injury showed improved cognitive performance 7 days post-injury, but the animals administered the inhibitor at 18 h post-injury did not. This suggests a very narrow window for TNF-targeting therapeutics after brain injury. Additional investigation showed that mice injected with TNF-inhibitor at 1 h showed fewer apoptotic neurons and less astrogliosis at 72 h post-injury in both the cortex and dentate gyrus. This study defined a 12 h window after an injury during which the negative effects of TNF may be attenuated, and points towards a tentative link between TNF and prolonged astrogliosis and neuronal death.

### Interleukin-10

IL-10 is an established anti-inflammatory cytokine with potent immunomodulatory effects. Knoblach and Faden [73] examined the effect of IL-10 using diverse administration paradigms in relation to TBI. When administered to rats intravenously (*iv*) at 30 min before and 60 min after a lateral fluid percussion (LFP) injury model, IL-10 resulted in superior neurological recovery after brain injury. This administration strategy also decreased the amount of IL-1β and TNF in the brain. When administered subcutaneously (*sc*) at 10 min, 1, 3, 6, 9 and 12 h after trauma, neurological recovery was improved at 7 days but the effect diminishes at 14 days. Interestingly, IL-10 showed no effect when administered by intra-cerebroventricular (*icv*) injection, suggesting the beneficial effects are mediated via the peripheral immune system. Kline et al. [74] found that after CCI, whilst systemic administration of IL-10 decreases the number of neutrophils hoarding in the parenchyma, it did not advance behavioural effects and reduced the neuroprotective effect of hypothermia.

It has been shown that interleukin-10 elevated in brain injury patients [45, 75–77], and has also been associated with unfavourable outcome and mortality [49, 76, 77]. Despite this, Chen et al. [41] uncovered the role of IL-10 in conferring neuroprotection with hyperbaric oxygen (HBO) treatment. They found that the protective effects of HBO in TBI included decreased lesion volume and oedema, improve cognition and diminishing pro-inflammatory cytokine production in the cortex following controlled cortical impact in wild-type mice vs IL-10-knockouts. This led to a shift from apoptotic to cell survival traits and superior BBB integrity. The link between IL-10 (and other cytokines) and poor outcome may be confounded by the widespread upregulation of cytokines after TBI.

### Transforming growth factor β

TGF-β, a 28-kDa dimeric protein, consists of two 14-kDa subunits and is produced by various cell types including T-cells and monocytes [78, 79]. TGF-β is pleiotropic in its action. It can restrict the growth of many cell types and also induce the secretion of the extracellular matrix. It can also antagonise many immune responses such as T cell and macrophage activation. Transforming growth factor-β rises acutely in the serum and CSF of TBI patients [80]. In several TBI models, TGF-β signalling mediators are upregulated [53, 81, 82]. Zhang et al. [53] showed that after weight drop TBI, TAK1 (transforming growth factor beta-activated kinase 1) increased in expression and was detected in cortical neurons as well as in astrocytes. Inhibition of TAK1 signalling diminishes NF-κB activity and inflammatory cytokine release which, in turn, improves the neuronal survival and motor functions. TGIF (a transcriptional co-repressor of TGF-β) can inhibit transcriptional activation of TGF-β. After knocking down TGIF levels in the brain by small hairpin RNA (ribonucleic acid), Chio and colleagues discovered that the drop in TGIF levels led to a reduction in infarct volume and microglia cell number around the region of induced TBI along with the noticeable modification in microglial morphology [81]. Chio et al. [81] found that knocking down TGIF improves motor function through 2 weeks post-injury. These data prove that mediators of TGF-β signalling can have important inflammatory consequences.

### Blood-brain barrier

The BBB is a complex structure that is involved in the pathogenesis of brain injury. It is formed by the neurovascular unit, the conjunction of cerebrovascular endothelial cells, pericytes, astrocytes and the basal lamina [83]. The BBB firmly regulates the exchange of all substances between plasma and the brain interstitium except small lipophilic molecules. Ion homeostasis and uptake of small molecules into the brain (e.g. glucose and amino acids) is conducted through specific endothelial membrane channels and solute carriers. Larger proteins and peptides are transported by endo-/trans-cytosis pathways within caveolae and clathrin-coated microvesicles. Notably, paracellular diffusion is greatly restricted by tight junctions amongst the apical regions of adjacent endothelial cells [83].

**Fig. 2:**
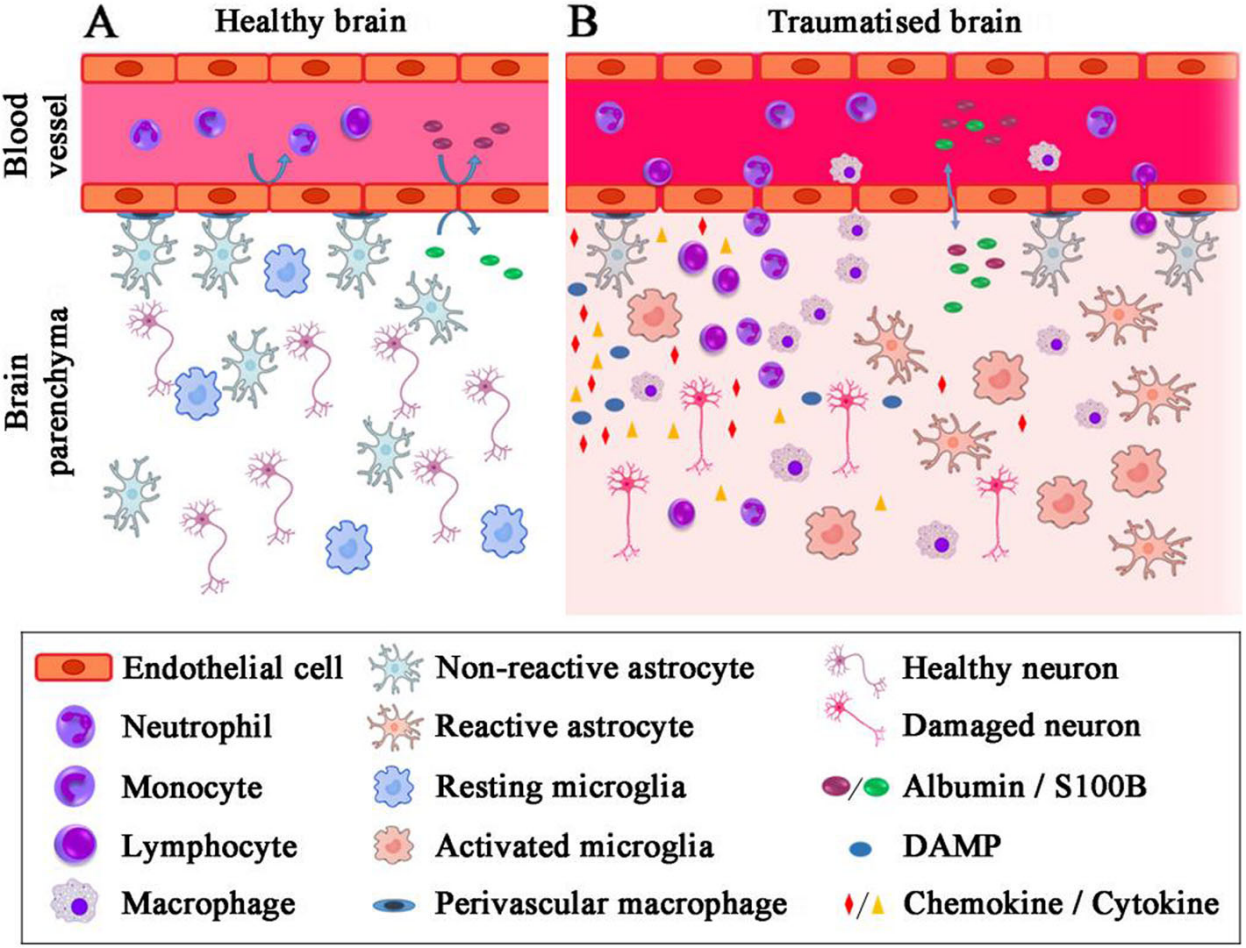
Cellular infiltration after TBI. **a** In healthy brain, the functional unit constituted by firmly coupled endothelial cells and astrocytic end-feet frame the blood-brain barrier (BBB). Immune cells flow unreservedly in the blood vessel, and in the brain parenchyma, blood-borne and brain-borne proteins cannot pass into the other compartment, resting microglia survey the intact brain ecosystem. **b** Following TBI, the BBB disrupted/leaked activating the endothelial cells. The tight junctions between endothelial cells perish. This allows immune cells to adhere at the blood vessels lamina and then transmigration to the brain parenchyma. Specific brain proteins (e.g. S100B) are released into the blood according to their concentration gradient in exchange, serum protein enters the brain parenchyma which has been demonstrated in the cartoon. Now, microglia switch from their resting state to an activated state, embracing a phagocytic phenotype and secreting pro-inflammatory proteins. Upregulation of adhesion molecules in cerebral vessels and production of chemokines by activated microglia and astrocytes finally cause blood leukocytes to migrate into the brain parenchyma where monocytes are suggested to cause additional damage to the brain

The brain is a highly vascularized organ for the efficient intake of oxygen and nutrients. Approximately 20% of the body’s total energy consumption takes place here [84]. In continuous and close contact with neurons and glia, the BBB is one of the most vital sites controlling the CNS microenvironment and homeostasis. It fulfils two main functions: (i) a physical barrier and (ii) a selective exchange barrier. Whilst the BBB has long been seen as a firm wall protecting the CNS, recent evidence reveals that this barrier is a lot more plastic and pliable than previously assumed. For decades, the immune privilege of the CNS perceived as an absence of immune system inside the CNS, and BBB was considered as the only barrier separating the CNS from the peripheral immune system and so preventing entry of infectious agents and immune cells [85]. Widespread work in the last decade unravelled the presence of a very specialised intrinsic innate immune system in the CNS [86]. This was supplemented by several observations showing that BBB is not just a passive barrier from an immunological point of view, but rather plays an active role in the immune response of the CNS [87]. Trauma to the brain results in disruption of the BBB, assisting recruitment of circulating neutrophils, macrophages and lymphocytes to the injured site [88]. The increase of systemic immune cells within the brain parenchyma has been reported in human TBI as well as animal models of brain trauma [89]. These cells release inflammatory mediators that direct glia and immune cells to the injury site. In addition to the infiltrating immune cells, the activation of resident microglia plays a major role in response to brain injury [90] (Fig. 2).

### Meninges

During inflammation, the entry of immune cells to the CNS parenchyma is secondary to their infiltration into the meninges [91]. Therefore, it is important to identify the mechanisms by which immune cells cross the meninges. Two plausible routes might explain how immune cells access the meninges: (i) through the meningeal blood vessels or (ii) via the choroid plexus (CP). The choroid plexus is located within each ventricle of the brain and is composed of epithelial cells surrounding the capillaries and stromal cells. The endothelial cells in the choroid plexus are fenestrated in the CNS [92]. The role of choroid plexus epithelial cells is to produce cerebrospinal fluid (CSF) by selectively extracting blood constituents, resulting in the highly vascularized choroid plexus, allowing the presence of many immune cells. However, this does not necessarily mean that cells can penetrate the epithelial layer and thus gain entry into the meningeal spaces or CSF. To access the meninges, immune cells from blood vessels supplying the choroid plexus need to cross the endothelial barrier and then the choroid plexus epithelial cell barrier with tight gap junctions to enter the CSF. For a cell to penetrate the meningeal vessels and enter the CSF, it has to cross the blood-meningeal barrier (BMB). The BMB differs from the BBB (blood-brain barrier) and lacks some components such as astrocyte end-feet, thus making it easier for cells to penetrate [93, 94].

A recent study suggests that meningeal blood vessels recruit T cells into the meningeal spaces [91]. Through activation of T cells in the meninges and their detachment are probably necessary for the cells to access the parenchyma; the route from the meningeal spaces/CSF to the parenchyma is not well understood. Meningeal cells can transmigrate across pia mater to reach the parenchyma [95], but the mechanisms guiding such a process are still unclear. Under neuro-inflammatory conditions, the gradient of chemokines produced and induced by meningeal immune cells might result in the transmigration of immune cells across the BBB [96].

## Cellular subtypes

### Neutrophils

Neutrophils are the most prevalent leukocytes in the blood, generated at a rate of ~ 2 × 10^11^ cells/day within the bone marrow [97]. In response to inflammation, when neutrophils come in contact with endothelium they get activated and express integrins. Integrins, in turn, bind adhesion molecules on the surface of the neutrophil to form contacts with the endothelium. Following this, integrins expressed on neutrophils bind to intracellular adhesion molecule (ICAM-1 and -2) to form a firm adhesion [98, 99]. Once a strong bond is established, trans-endothelial migration occurs which allows entry of the neutrophils into the damaged tissue where they perform phagocytic and clearance functions [100]. In animal models, the earliest cells that enter the site of neural injury are neutrophils, which appear within minutes and peak 2 h later in the subarachnoid and subdural spaces [98, 101]. Infiltration into the brain parenchyma peaks 24 to 48 h in the course of the inflammatory cascade after cortical injury [98, 102] then decreases substantially over an ensuing 7 days [59, 103, 104].

Few studies have been conducted investigating neutrophil dynamics in clinical TBI as the cells are only present in the hyper-acute/acute phase. Histopathological analyses have shown they are also few and present only at the earliest stages of brain injuries. Notably, in brain tissue collected from post-mortem TBI, neutrophils can be regularly observed in the first hours following injury, though there is evidence that the cells can enter the brain in minutes [105]. A comprehensive study including brains from 305 post-traumatic autopsy cases found evidence of neutrophil infiltration in approximately 43% of samples from 5 min after injury [106]. These data suggest that neutrophil recruitment following injury is a significant early event in TBI pathogenesis.

#### Neutrophil toxicity

Neutrophil infiltration also appears to be a mechanism for protecting the brain in injury states although neutrophils are toxic to neurons. Neutrophils are vigorously recruited to the damaged brain when there is potential infection: strong neutrophil infiltration is observed in the LPS (lipopolysaccharides)-injected brain or contusion-injured spinal cord, but not in the ATP (adenosine triphosphate)-injected brain or laceration-injured spinal cord [107–109]. Neutrophils are the first-responder cells that enter damaged tissue to protect the body from potential infection. Thus, they express cytotoxic inflammatory mediators, like iNOS (inducible nitric oxide synthase) and MPO (Myeloperoxidase). Neutrophils also express cytotoxic inflammatory mediators in the injured brain [109].

#### Blood-brain barrier break down

Neutrophils can break down the blood-brain barrier by releasing metalloproteinases, proteases, TNF and ROS. After brain injury, the release of inflammatory mediators can assist this process by inducing a hyper-activated state that permits neutrophils to break the BBB and enter the CNS [110]. On arrival, neutrophils have the power to inflict neuronal cell death exploiting the same soluble mediators that break down the BBB [111]. Liao et al. [106] revealed that neutrophils are the most abundant circulating cells after TBI and cause amplified expression of oxidative enzymes indicative of activation [64]. Both in the immediate aftermath of TBI as well as after some days following injury, studies have shown that relative to healthy control values, both the absolute number and frequency of circulating neutrophils are significantly increased, with Rhind and coworkers [112] reporting a 4.5-fold elevation in neutrophil numbers as early as 3 h post-TBI.

#### Neutrophilia after TBI

This immediate neutrophilia is thought to result from TBI-induced increases in serum catecholamines and glucocorticoids. A surge in catecholamines will trigger the entry of marginated neutrophils into the circulation, whilst a rise in glucocorticoid levels increase the size of the peripheral neutrophil pool by stimulating their release from bone marrow stores and by extending their lifespan and preventing circulating neutrophils from returning to the bone marrow for clearance [113].

#### Neutrophil and ROS generation

In contrast to the enhanced ROS generation that characterises the initial response to TBI, neutrophils exhibit impaired ROS generation in the days following injury [114]. In a study of hospitalised TBI patients with moderate or severe brain trauma, Marks et al. [115] found that neutrophil ROS production on day 9 post-injury was significantly lower than that of healthy age- and sex-matched controls. Given that peak incidences of infection in hospitalised TBI patients occur 5–11 days after injury, and neutrophils are the first line of defence against rapidly dividing bacteria, fungi and yeast, then impaired ROS generation may be one mechanism underlying the increased susceptibility of hospitalised TBI patients to infection [116]. Keeping this in mind, a comparison of ROS generation between neutrophils isolated from non-infected and infected TBI patients revealed a significantly greater percentage of ROS producing cells in the uninfected group on day 6 post-injury [115].

### Monocytes

Monocytes are a heterogeneous population of blood-borne leukocytes comprising about 5–10% of circulating immune cells. Liao et al. [64] showed that brain injury leads to an increase in the absolute number of circulating monocytes [112]. Their study revealed that the increase in monocytes is 2.7-fold higher relative to healthy controls within 24 h of injury. Post-TBI, this increase in monocyte number reflects the augmentation of specific subsets outline in the surface phenotype of circulating monocytes. In the injured brain, the role of monocytes is similar to microglia. Monocyte activation is classified as classical (bactericidal) and alternative (reparative). When monocytes are exposed to classical activators (e.g. IFN-γ, CCL2) they produce significant amounts of cytotoxic inflammatory mediators including reactive oxygen species (ROS) and TNF. In contrast, monocytes activated by interleukins (IL-4 and IL-13) express reparative genes, comprising mannose receptors [117]. However, according to Chen et al. [118], monocytes invading the brain parenchyma in response to the traumatic brain injury have a detrimental effect on neuronal survival and functional recovery. The influx of these inflammatory cells is driven by monocyte chemo-attractants (especially CCL2) whose synthesis is rapidly increased after brain injury [26, 119]. In a 2012 study, Szmydynger-Chodobska et al. [120] demonstrated that neuro-trauma also results in a rapid increase in the production of CCL2 by the lateral ventricle choroid plexus (CP) located ipsilaterally to injury. The increase in choroidal CCL2 synthesis was not associated with post-traumatic accumulation of monocytes in the choroidal tissue but resulted from the production of CCL2 by the choroidal epithelium [120]. Their results were consistent with a previous study by Colotta et al. [121], which showed that although monocytes can produce CCL2 in response to pro-inflammatory mediators; these inflammatory cells were rarely found in the ipsilateral choroid plexus (CP) at 6 h post-TBI. An increase in choroidal production of CCL2 was associated with a significant elevation of CCL2 concentration in the CSF, which was comparable to the levels of chemokine found in the CSF of patients with severe TBI [26].

In the contusion-injured spinal cord, Kigerl et al. [122] reported that monocytes alternatively activated in vitro change to classically activated cells about 7 days after transplantation. However, it is unlikely that monocytes are classically activated and/or change their phenotype from alternative to classical activation since debris and dead cells disappear and a cavity is formed after monocyte infiltration [123], indicating that monocytes are alternatively activated and phagocytose dead cells and debris. Furthermore, damage does not increase further during or after monocyte infiltration [124], an outcome that would not be expected if monocytes were classically activated and produced cytotoxic mediators. Damage increases between 12 to 24 h after contusion-induced spinal cord injury but does not increase thereafter [124]. Jeong et al. [125] also reported that monocytes infiltrating the injured brain and spinal cord express alternative activation markers [108]. Even in LPS-injected brains, microarray analyses have revealed that repair-related genes, including those for proliferation, wound healing and phagocytosis, increase at times corresponding to monocyte infiltration [125]. More importantly, the damaged brain is repaired after monocyte infiltration, as evidenced by the fact that astrocytes, blood vessels, oligodendrocytes, myelin and neurites reappear and fill the damaged core [125]. Monocytes appear to produce certain chemokines that recruit astrocytes which, in turn, extend their processes towards monocytes during the recovery phase. For this reason, the entry of monocytes into the brain is physiologically relevant for the repair of damaged tissue.

After completing this reparative role, infiltrating monocytes disappear from the damaged brain within 1 or 2 months via two different pathways. Most monocytes die in the brain about 5 days after infiltration [125], whilst some monocytes differentiate into resident brain microglia [126], providing a source of monocyte-derived microglia to replenish areas in the damaged core deprived by the death of resident microglia [108] (Fig. 1b).

### Microglia

Microglia are highly dynamic CNS resident innate immune custodians. These cells are derived from embryonic yolk-sac macrophages that migrate to the brain upon the development of the cerebral vasculature [127, 128]. Microglia participate in a variety of homeostatic CNS functions, like synaptic plasticity and learning [129], and often respond rapidly to any inflammatory events that occur within the CNS parenchyma [128]. Activated microglia/macrophages release pro- and anti-inflammatory factors that send signals to resident and peripheral cells to promote/resolve the inflammatory response to trauma. Chronically activated microglia/macrophages have been found in rodent models and humans after TBI [130–133] and are considered to be one of the hallmarks of unresolved inflammation that may have long-term repercussions [134, 135].

Peripheral microglia/macrophage phagocytic cells are the first-line innate immune cell of the brain and make up 10% of total brain cells [136]. Following brain damage, these cells become quickly activated by purinergic signalling through the P2Y family of receptors. This leads to cell migration from the outer area (< 75 μm in radius) into the inner area (< 40 μm in radius) of injury [29]. Macrophages migrate towards the damaged site whilst adjacent astrocytes move further away and extend their processes. Furthermore, the release of ATP from damaged cells, DAMPs and activation pathways influence immune activation after brain injury. High mobility group box-1 (HMGB1) protein was found to translocate from the neuronal nucleus to the cytoplasm in the early hours following brain injury and localised to microglia at a later stage [137].

Due to similar origin, it is difficult to detect the presence and functions of activated microglia versus peripheral blood-borne macrophages that infiltrate the brain following injury. Jin et al. [104] classified two subsets of cells based on their degree of binding to CD45; CD45High/CD11b + ‘macrophages’ and CD45Low/CD11b + ‘microglia’. Based on these measures, CD45High/CD11b + cells peaked within 1 day following controlled cortical impact and remained elevated at 3 days before reaching control levels. Jin et al., on the other hand, demonstrated that microglia increase in numbers to a peak at day 7 post-injury then decrease over the next week before increasing again through 28 days [104]. These dynamics for microglia activation are similar to those proposed by Loane and Byrnes [90], who also cited evidence that a second peak occurs between 30 to 60 days (Fig. 1a).

#### Microglial activation/polarisation

**Fig. 3:**
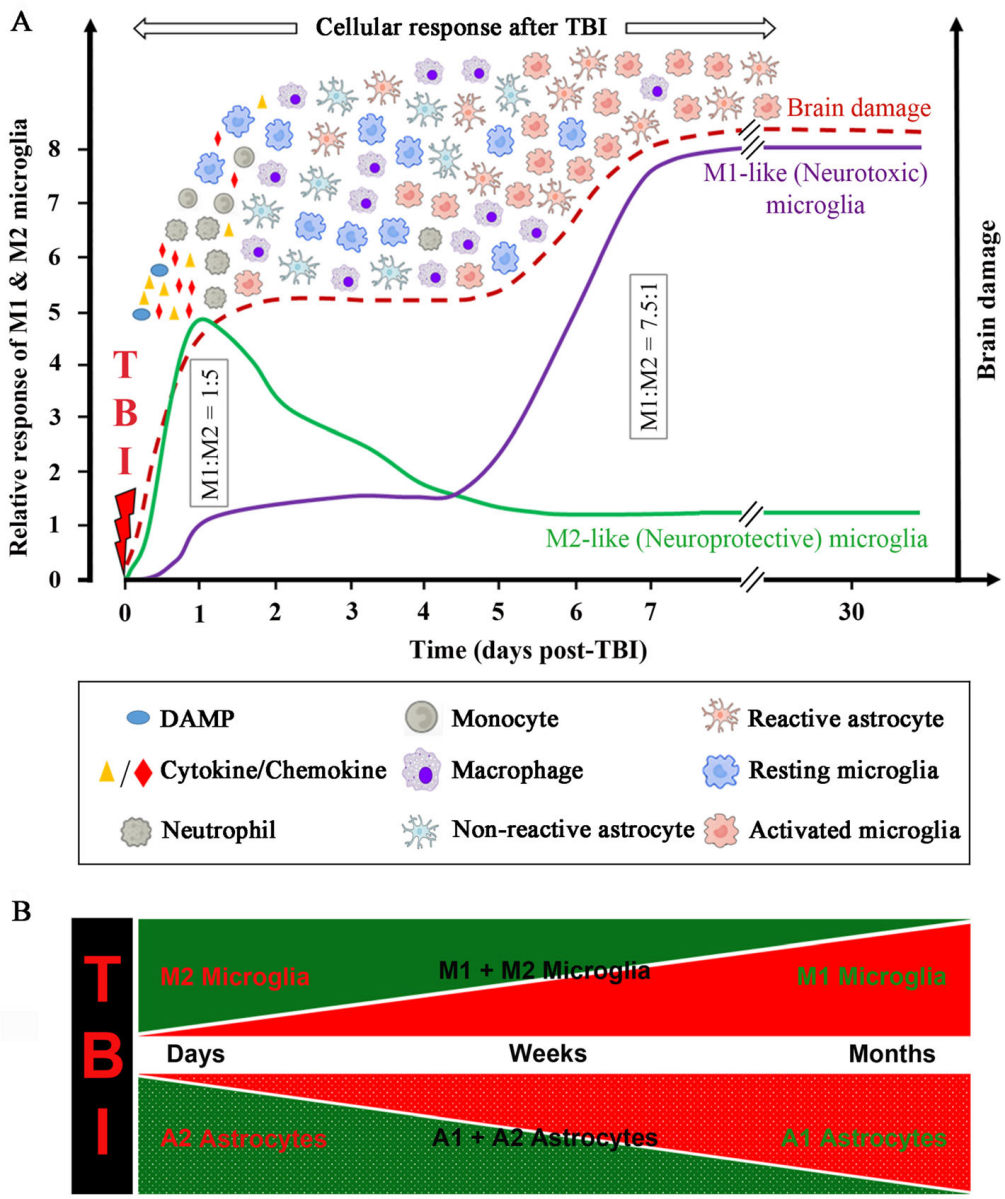
Graphical representation of the cellular response and brain damage post-TBI, including the proposed microglia (M1 and M2) and astrocytes (A1 and A2) dynamics over time. **a** Injury to the brain may cause cell membrane disruption, vascular rupture and BBB damage. This leads to the release of DAMPs, cytokines, chemokines immediately after injury and peaks within minutes to hours and continue to release by the damaged tissue and infiltrating cells. This causes the activation of microglia and astrocytes and recruitment of circulating immune cells. These immune responses are largely overlapped (Fig. 1). The inflammatory response is the key to debris clearance, repair and regeneration post-TBI. But skewed inflammation might lead to secondary brain injury. The role of microglial activation is increasingly recognised as both a critical pathological mechanism and therapeutic target. Specifically, there is a shift from a dominance of M2 (neuroprotective) microglia into a preponderance of M1 (neurotoxic) microglia [141]. M1:M2 microglia population ratio shifts from 1:5 (day 1) to 7.5:1 (day 7) following injury [142]. Also, Wang et al. [143] whilst working with CCI mice model discovered that the phenotypic ratio of M1 and M2 at 48 h (day 2) is 1:3 and the M2-like microglia/macrophages peaked at day 5 but decreased rapidly thereafter. **b** Both microglia and astrocytes are highly sensitive to their environment. Unlike other inflammatory cells, astrocytes and microglia are in a constant dynamic mode. Their subtypes will shift with time and space, and with other unknown variables. Microglia and astrocytes so far have been shown to exist in two distinct reactive states (Microglia, M1-neurotoxic and M2-neuroprotective; Astrocytes, A1-neurotoxic and A2-neuroprotective). Considering these two states, it is possible that they exist as a continuum, with a heterogeneous mixed population in the middle. It should be noted that this classification is somewhat limited because microglia/macrophages can exhibit more than two canonical polarisation states [144]. In much a similar way, reactive astrocytes might have more than two polarised states. The heterogeneity of reactive microglia and astrocytes need to be investigated thoroughly

It is now established that there is significant heterogeneity of microglial activation in the brain. Microglia have notable plasticity that allows them to competently respond to the signals from the microenvironment and change their phenotype and function accordingly [117, 138]. This process is called microglial polarisation which allows the adaptive responses of innate immunity to succeed. Two conceptual microglial polarisation states have been defined, termed M1 and M2, which denote both ends of a continuum of functional microglia/macrophage activation [117, 138]. The dual characters of polarised microglia have been identified in several CNS conditions including stroke [139], multiple sclerosis [140] and spinal cord injury [122]. Infiltrating microglia/macrophages can assume neurotoxic (M1) or neuroprotective (M2) phenotypes following traumatic brain injury. Jin et al. [104] suggested that the brain experiences a bimodal surge in the microglial number after brain injury (Fig. 3).

However, despite their different ontogeny, the specific roles of polarised microglia (M1 and M2) in brain injury progression and repair have not been explored fully. We studied the cytokine response to human traumatic brain injury and noted that TNF which is the marker of M1-type microglia appears/peak earlier than the IL-10 (M2-type microglia marker) [28]. According to Ransohoff [145], the approval of this phenomenon was just an attempt to simplify the interpretation of data at a time when the evolution and functional understanding of microglia was beginning to be understood. Henry et al. [146] demonstrated that temporary diminution and subsequent re-population of microglia during the chronic phase of experimental brain injury decreases chronic neuroinflammation, advances neurological recovery and ameliorates neurodegeneration. These findings suggest that the therapeutic window for targeting chronic neurodegeneration and refining functional recovery after TBI may be more prolonged than traditionally believed [147].

### Astrocytes

Astrocytes surrounding the injured region respond to cytokines and chemokines which in turn can damage the local neurons, and activate the surveillance microglia. Astrocytes are activated by cell debris and inflammatory mediators present in the milieu and can be identified as GFAP+ (glial fibrillary acidic protein) or more appropriately as ALDH1L1+ (aldehyde dehydrogenase 1 family, member L1) cells [148].

The astrocyte was originally thought to have merely a supportive role for brain homeostasis hence the etymology of glia as ‘brain glue’. It has long been recognised that astrocytes have a wider functional part to play, being responsible for upholding water and ionic balance, the integrity of the blood-brain barrier and maintenance of neurotransmitter homeostasis. Post TBI, astrocytes become reactive and undergo a phenotypic change including cellular enlargement, an extension of its processes, and enhanced expression of GFAP. Following brain injury, astrocytes perform critical functions but it is not clear whether these reactive astrocytes are beneficial/detrimental to the disease pathology [149]. Clear evidence supporting a neuroprotective role of reactive astrocytes post-TBI includes reduced neuronal staining with NeuN or cresyl violet that correspond to areas lacking GFAP+ cells [150]. Kelso and Gendelman [151] also found a significant increase in CD45+ cells in regions lacking reactive astrocytes, suggesting infiltration of inflammatory phagocytic cells. Astrocytes produce soluble factors such as TGF-β and prostaglandins which might inhibit microglial activation [152–156]. Ji and coworkers [157] have demonstrated that uncharacterised soluble factor(s) released from astrocytes induce the expression of antioxidant enzymes, which in turn inhibit microglial activation. In the injured brain, astrocytes rapidly-produce anti-inflammatory molecules, which could be one of the mechanisms for reversing microglial activation in TBI. Furthermore, astrocytes support neuronal survival by providing growth factors and nutrients to neurons and maintaining homeostasis of extracellular fluid by boosting glutamate and potassium uptake. Jeong et al. [125] reported that the functional loss and/or death of astrocytes preceded secondary neuronal death in spinal cord injury. Based on these findings, Jeong et al. [125] propose that the loss of astrocyte function might be caused by secondary brain injury.

We recently reported how in vitro human astrocyte cultures react to cytokines across a concentration range that approximates the outcome of human TBI [19]. Experimentally, enriched cultures of human-induced pluripotent stem cell (iPSC)-derived astrocytes were exposed to IL-1β (1-10,000 pg/mL), IL-4 (1-10,000 pg/mL), IL-6 (100-1,000,000 pg/mL), IL-10 (1-10,000 pg/mL) and TNF (1-10,000 pg/mL). Cultures were fixed and immune-labelled at different time points (1, 24, 48 and 72 h), and the secretome was analysed using a human cytokine/chemokine 39-plex Luminex assay [19]. Results showed that the exposure of IL-1β causes the most profound downstream response and TNF, IL-6 had intermediate responses, whilst IL-4 and IL-10 only led to weak responses over time and/or increasing concentrations. We also reported that expression of IL-1β, IL-6 and TNF receptor mRNA was higher in astrocyte than in neuronal cultures. Various secreted cytokines had temporal trajectories, which corresponded to those seen post-human-TBI.

#### Astrocyte activation/polarisation

Analogous to microglial polarisation states, it has been suggested that neuroinflammation can stimulate two types of astrocytes, A1 and A2 (Fig. 3b). Liddelow and Barres [158] recently postulated that astrocytes can switch in a stimulus-specific manner towards a classical pro-inflammatory (harmful) A1-phenotype or an alternative anti-inflammatory (beneficial) A2-phenotype. This mirrors the classical and alternative states seen in microglia. Although we have noted earlier that microglial signalling regulates astrocyte functions [159], both the stimulus and the physiological mechanisms leading to the transformation of astrocytes remain unclear. Transcriptome-wide analyses of astrocytes showed that A1-reactive (neuroinflammatory) astrocytes stimulate many complement cascade genes that have been previously known to be detrimental to synapses, implying that A1 astrocytes might have harmful effects. On the contrary, A2 reactive astrocytes stimulates numerous neurotrophic factors promoting growth and survival of neurons, and thrombospondins, which in turn promotes synaptic repair. This stimulation suggests that A2 astrocytes might have beneficial functions [160, 161].

Recently, Neal et al. [162] discovered that PK2(prokineticin 2)-induced astrocyte reactivity leads to an increase in antioxidant and anti-inflammatory proteins with increasing glutamate uptake resulting in decreased inflammatory factors. Furthermore, Ma and colleagues [163] whilst working on SAH (subarachnoid haemorrhage)-induced early brain injury added that administration of rPK2 (recombinant PK2) or augmenting PK2 expression may stimulate a modulation of astrocytic polarisation towards a more protective (A2-type) phenotype. On the other hand, Miyamoto et al. [164] suggested that prolonged cerebral hypoperfusion leads to impaired oligodendrocyte maturation which, in turn, increases A1-type astrocytes number. Whilst working with the rat primary cells cocultured with a nonlethal concentration of CoCl_2_, they observed that at 28 days after hypoperfusion, the number of astrocytes increased and oligodendrocyte decreased. Interestingly, increased astrocytes were mainly A1-like astrocytes; however, the number of A2-like type decreased. Thus, ligands central to either the A1-reactive (proinflammatory) state or A2-reactive (protective) state may serve as potential therapeutic agents for a number of neurodegenerative diseases in the future.

### Neurons

Neurons present in the injured region experience mechanical forces at dendrites, cell body, axon and are recognised in tissue sections as NeuN+ cells. Force transmission to the head with or without direct impact leads to it stretching, bending or shearing. The axonal injury associated with TBI can be seen at deep sites at the junction between grey and white matter, particularly in the corpus callosum. In addition to primary injury, the secondary injury phase also causes damage to the neurons by excitotoxic compounds and inflammatory mediators present in the extracellular space. Interaction of neurons with microglia and astrocytes is a key factor for inhibiting unwarranted microglial activation [165]. Neurons inhibit microglial inflammatory responses via expression of ligands (e.g. CD200 and fractalkine), whose receptors are expressed in microglia [166]. Consistent with this, Cardona and colleagues proved that brain inflammation is significantly enhanced in fractalkine receptor-knockout mice [167]. Therefore, neurons inhibit microglial activation in the intact brain [168].

In a cell culture model of neuronal reactivity, one of our recent papers, we witnessed the production of different patterns of cytokines in enriched human cortical neuronal cultures after cytokine induction which might alter the pathophysiological processes potentially in both beneficial and detrimental ways in the in vivo state [18]. Our main focus was to elucidate the secondary cytokine responses to primary known pro-inflammatory cytokine exposure, and we validated the data clinically against cytokine patterns seen post-TBI. We overcome the inaccessibility to human neuronal models by exploiting directed differentiation of human pluripotent stem cells, which represent a reductionist, and clinically relevant and reliable model system. Further, in the absence of glia, it has been shown that IL-1β is not directly toxic to neuronal cells, suggesting that neurons may not be the primary target of IL-1β [169, 170]. Deshpande et al. [169] in their paper showed induced neuronal death in IL-1β treated astrocytic-culture supernatant on a human foetal neuron culture. Furthermore, in human foetal neuronal-glial co-culture model, IL-1β and TNF did not show neurotoxic effects in isolation but triggered neuronal death in combination [171]. Likewise, induction of TNF produces the synergistic neurotoxic effect of IL-1β and IFN-γ. To sum up, IL-1β exposure did not produce the same degree of cytokine generation as IL-6 and TNF, which might be due to the enriched neuronal monoculture paradigm.

### Adaptive immune response

The adaptive immune response consists of two parts: (i) humoral immunity and (ii) cell-mediated immunity. Humoral immunity is mediated by B-lymphocytes and is responsible for the production of antibodies following activation by an antigen. Cell-mediated immunity is primarily directed by activated memory effector T-lymphocytes or regulatory cells and can have destructive or homeostatic effects. These responses can be activated as a result of protein modifications that result from brain injury and lead to chronic neurodegeneration [172, 173].

#### B cells

B-lymphocytes arise from progenitors in the bone marrow and enter the circulation, ultimately forming nests in the spleen and lymph nodes. Following activation by a pathogen, they differentiate into plasma cells whose primary function is to produce antibodies. Involvement of B cells following brain injury has not been studied in great details as compared to the other aspects of immunity. OX33, a B cell marker has been reported 4 to 6 days after experimental injury [174]. Meissner A et al. [175] showed that a chemokine associated with B cell chemotaxis (CCL20) is elevated within 4 h and persists out to 3 days following experimental brain injury (CCI) [176]. Few clinical studies have also been conducted to determine the effect of brain injury on B cell populations. Mrakovcic-Sutic I et al. [177] investigated B cell (CD5+/CD19+) populations in the peripheral blood of 20 patients with severe TBI and found no significant difference when compared to healthy controls.

Until recently, TBI was thought to have no impact upon B-cell biology, an idea based on a handful of experimental studies that had shown no alterations in the occurrence, number or proliferative capacity of B cells following brain injury [114, 177–180]. But more recently, studies performed by two groups of scientists [181, 182] demonstrated that autoantibodies specific for CNS proteins were detected in the serum of brain injury patients, suggesting that tolerance is broken and B cells initiate an immune response against brain-derived antigens following injury. Marchi et al. [181] uncovered that serum levels of the astrocytic protein (S100B) were higher in subjects with experienced frequent sub-concussive head hits (SHH) accompanied by an elevated level of anti-S100B auto-antibodies. Increased S100B auto-antibody was linked with low cognitive functions, and these auto-antibodies reacted strongly towards epitopes of both the glial and neuronal cells. It was believed that the production of auto-antibodies against nervous system-residing proteins may serve as a risk factor for premature neurodegeneration in repeated SHH [181]. Similar to this, Zhang et al. [182] identified auto-antibodies against the astrocyte-residing transitional filament protein GFAP and its breakdown products obtained from patients following severe TBI in serum samples. These GFAP auto-antibodies, which were detected as quickly as 4 days post-injury and whose range positively correlated with GCS (Glasgow Coma Scale) scores suggesting a potential pathophysiological duty for B cells during the recovery phase of brain injury [182]. It is now established by different studies that in both acute and chronic post-TBI, AutoAb [GFAP] is useful to study the dynamic interactions amongst brain autoimmune mechanisms. The important new finding reported by Wang et al. [183] in their study suggested that in acute TBI, plasma AutoAb [GFAP] levels correlate with a history of past TBI exposure. Cox and colleagues [184] hypothesised that the exposure of lymphocytes to abnormally large quantities of myelin antigens would kindle proliferation of myelin specific lymphocytes. They validated a substantial increase in the number of myelin-specific auto-reactive lymphocytes, and the degree to which they multiply, after TBI. These data for the first time reported that autoreactive T cell responses directed at myelin antigens occur in human brain injury.

#### T cells

Brain injury (severe TBI) results in a significant decline in the percentage and an absolute number of circulating T lymphocytes [114, 185]. Mrakovcic-Sutic et al. [177] detected that this decline within 24 h of injury and on day 4 post-injury, is the result of a considerable reduction in both CD4+ T helper cells and CD8+ cytotoxic T cells. Presently, the mechanism(s) underlying TBI-induced drop of the circulating T-cell pool is not fully clear. After a series of in vivo experiments, Nakai et al. reported that administration of B2-adrenergic receptor (B2AR) agonists is followed by a rapid decrease of blood CD4+ and CD8+ T cells. With further investigations, they revealed that this lymphopenia was the result of B2AR stimulation inhibiting lymphocyte departure from lymph nodes [186]. Given that TBI results in elevated levels of circulating catecholamines [187, 188], then lymphocyte retention in lymph nodes may be one of the mechanisms which explain the significant reduction in circulating T cells that have been witnessed following brain injury.

Generally, human studies have supported the pre-clinical confirmation of T cell recruitment following brain injury. Holmin et al. [189] performed an analysis of brain biopsy and tissue collected from TBI victims at different time points post-injury. This study assessed the percentage of patients who had immunohistochemical evidence of inflammatory cells present in the tissue sample. The authors could not identify CD3+, CD4+ or CD8+ T cells in brain biopsies collected within 24 h following brain injury. However, all brain samples collected 3 to 5 days post-trauma had some expression of CD3+ and CD4+ cells, and 75% of the samples had CD8+ cells. Whilst this pattern supports pre-clinical evidence of late involvement, it should be noted that the injuries did not happen in the same area for all patients and that this study included only 12 patients, 4 of which were in the 3 to 5-day group [189]. One study investigating 56 post-mortem, TBI cases found CD3+ T cells in the lesion area after 4 days post-injury and persisted for at least 26 days [105], but they did not assess T cell subsets by pinpointing any other markers.

## Conclusion

Cellular infiltration in TBI is a complex process involving several types of cells and soluble mediators, including DAMPs, cytokines, chemokines, neutrophils, monocytes, microglia, astrocytes and neurons. Microglia continuously survey the brain to identify any structural abnormalities in neurons. In response to brain trauma, microglia at the injury penumbra quickly isolate the damaged core region and prevent the spread of the unsettled injury microenvironment to surrounding regions. Then, there are neutrophil infiltrates to protect the region from further infection. To repair the damaged brain, monocytes infiltrate the injured site and peak around 3-5 days post-injury. We suggest that the secondary injury (brain damage) that exacerbates acute injury may be caused by the secondary activation of M1-like (neurotoxic) microglia. Hence, a detailed understanding of cellular and molecular mechanisms, and implementation of reductionist models [18, 19] for the acute and chronic TBI phases, will serve to advance our understanding and management of TBI. A mechanistic understanding of primacy and roles of the various cells present in response to injury (which can be termed ‘the cellular phase’ of TBI) might prove transformational in identifying promising therapeutic targets within a wider time window for potential intervention in a majority of the frequently devastating cases of TBI.

## Data Availability

Not applicable.

## Abbreviations

A1: Neurotoxic astrocyte
A2: Neuroprotective astrocyte
ALDH1L1: Aldehyde dehydrogenase 1 family, member L1
ATP: Adenosine triphosphate
B2AR: B2-adrenergic receptor
BBB: Blood-brain barrier
BMB: Blood-meningeal barrier
CCI: Controlled cortical impact
CCL2: C-C motif chemokine ligand 2
CNS: Central nervous system
CP: Choroid plexus
CSF: Cerebrospinal fluid
CXCL10: C-X-C motif chemokine-10
DAI: Diffuse axonal injury
DAMPs: Damage-associated molecular patterns
ECF: Extracellular fluid
GFAP: Glial fibrillary acidic protein
GM-CSF: Granulocyte-macrophage colony-stimulating factor
HBO: Hyperbaric oxygen
HMGB1: High mobility group box-1
ICAM: Intracellular adhesion molecule
IFN-γ: Interferon gamma
IL: Interleukin
iNOS: Inducible nitric oxide synthase
iPSC: Induced pluripotent stem cells
*iv*: Intravenous
*icv*: Intra-cerebroventricular
JAK-STAT: Janus kinase-signal transducers and activators of transcription
LFP: Lateral fluid percussion
LPS: Lipopolysaccharides
M1: Neurotoxic microglia
M2: Neuroprotective microglia
MP: Mononuclear phagocytes
MPO: Myeloperoxidase
NeuN: Neuronal nuclei (antibodies)
PCA: Principal component analysis
PK2: Prokineticin 2
RNA: Ribonucleic acid
ROS: Reactive oxygen species
*sc*: Subcutaneous
SHH: Sub-concussive head hits
TBI: Traumatic brain injury
TNF: Tumour necrosis factor
TGF-β: Transforming growth factor-beta
